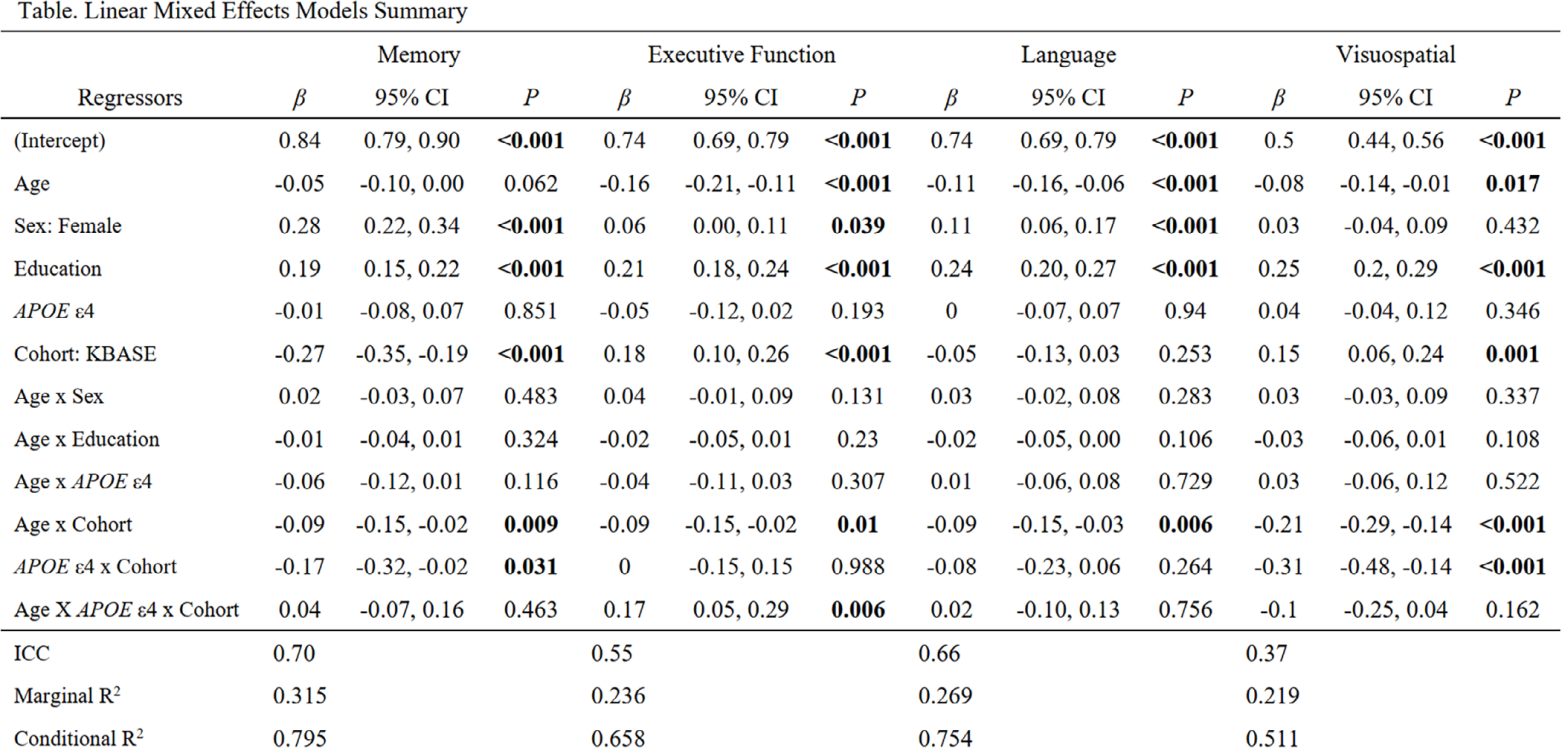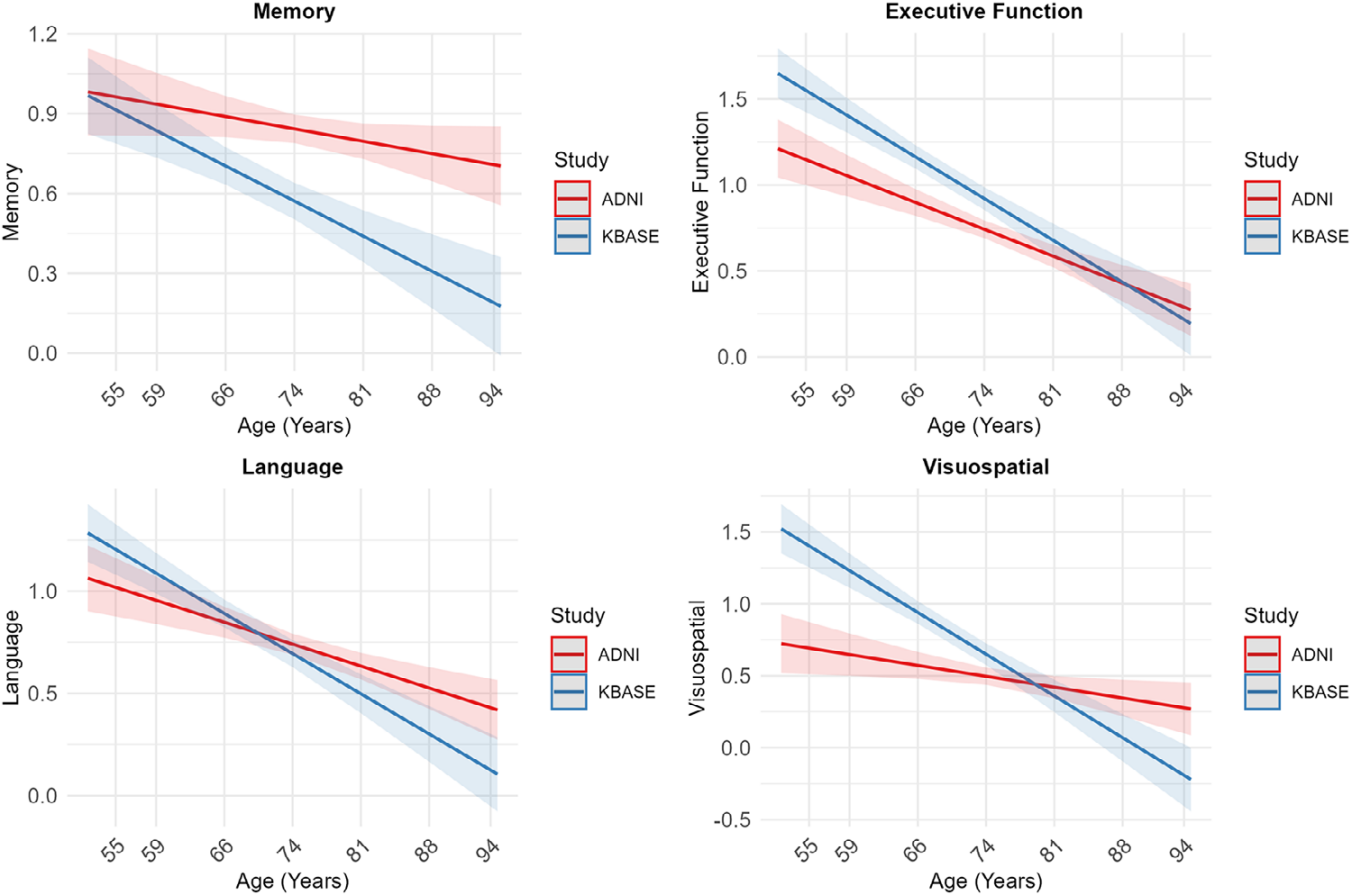# The Differential Impact of *APOE ε4* on Normative Cognitive Aging in Research Samples from the United States and Korea

**DOI:** 10.1002/alz70857_107664

**Published:** 2025-12-24

**Authors:** Joshua M. Garcia, Dahyun Yi, Min Soo Byun, Dong Young Lee, Kwangsik Nho, Shannon L Risacher, Andrew J. Saykin, C. Elizabeth Shaaban, A. Zarina Kraal, Martine Elbejjani, Diefei Chen, Jeremy A. Tanner, Adam M. Staffaroni, Paul K Crane, Luis D. Medina, Kacie D Deters

**Affiliations:** ^1^ University of Houston, Houston, TX, USA; ^2^ Institute of Human Behavioral Medicine, Medical Research Center, Seoul National University, Seoul, Korea, Republic of (South); ^3^ Department of Neuropsychiatry, Seoul National University Hospital, Seoul, Korea, Republic of (South); ^4^ Department of Psychiatry, Seoul National University College of Medicine, Seoul, Korea, Republic of (South); ^5^ Indiana University School of Informatics and Computing, Indianapolis, IN, USA; ^6^ Center for Neuroimaging, Department of Radiology and Imaging Sciences, Indiana University School of Medicine, Indianapolis, IN, USA; ^7^ Indiana Alzheimer's Disease Research Center, Indiana University School of Medicine, Indianapolis, IN, USA; ^8^ Center for Neuroimaging, Department of Radiology and Imaging Sciences, Indiana University School of Medicine, Indianapolis, IN, USA; ^9^ Department of Medical and Molecular Genetics, Indiana University School of Medicine, Indianapolis, IN, USA; ^10^ Indiana Alzheimer's Disease Research Center, Indiana University School of Medicine, Indianapolis, IN, USA; ^11^ Center for Neuroimaging, Indiana University School of Medicine, Indianapolis, IN, USA; ^12^ University of Pittsburgh Alzheimer's Disease Research Center (ADRC), Pittsburgh, PA, USA; ^13^ Taub Institute for Research on Alzheimer's Disease and the Aging Brain, New York, NY, USA; ^14^ American University of Beirut, Beirut, Lebanon; ^15^ Department of Epidemiology, Bloomberg School of Public Health, Johns Hopkins University, Baltimore, MD, USA; ^16^ Glenn Biggs Institute for Alzheimer's & Neurodegenerative Diseases, University of Texas Health Science Center, San Antonio, TX, USA; ^17^ Memory and Aging Center, UCSF Weill Institute for Neurosciences, University of California, San Francisco, San Francisco, CA, USA; ^18^ Department of General Internal Medicine, University of Washington School of Medicine, Seattle, WA, USA; ^19^ University of California Los Angeles, Los Angeles, CA, USA

## Abstract

**Background:**

Normative cognitive aging and the influence of genetic risk factors on neurodegenerative disease processes differ between ethnic and racialized groups. The aims of this study were to examine differences in normative cognitive aging between research cohorts from the United States and Korea and evaluate the impact of *APOE ε4*.

**Method:**

We estimated normative cognitive aging patterns among non‐Hispanic white participants in United States (ADNI, *N* =  609; Age_mean_=73±6; % Female=54; Years of education = 16.7±2.5) and Korean (KBASE, *N* = 245; Age_mean_=68±8; % Female=50; Years of education = 12.3±4.6) cohorts who remained free of cognitive impairment over 4‐years of follow‐up using linear mixed effects models by age. Harmonized data were leveraged to estimate cognition by age across domains of memory, executive function, language, and visuospatial abilities. Linear mixed‐effects models with a random intercept by participant and fixed effect interactions of age with sex, education, *APOE ε4* (excluding ε2/ε4 carriers), and cohort (ADNI vs. KBASE) were specified for each domain; a three‐way interaction between age, *APOE ε4*, and cohort was also specified.

**Result:**

Normative aging patterns demonstrated higher intercepts for executive functioning (*β*[95%CI]=0.18[0.10,0.26]) and visuospatial (*β*[95%CI]=0.15 [0.06,0.24]) domains in KBASE compared to ADNI, with a lower intercept for memory (*β*[95% CI]=‐0.27[‐0.35,‐0.19]), and no difference in the intercept for language (*β*[95%CI]=‐0.05[‐0.13,0.03]). Participants in KBASE exhibited steeper age‐related declines across all domains of cognition (*β_range_* = ‐0.21,‐0.09). *APOE ε4* carrier status was associated with lower memory (*β*[95%CI]=‐0.17[‐0.32,‐0.02]) and visuospatial (*β*[95%CI]=‐0.31[‐0.48, ‐0.14]) intercepts and a slower rate of age‐related decline for executive function (*β*[95%CI]=0.17[0.05,0.15]) in KBASE, but not ADNI (Table, Figure). Main effects of interest were broadly consistent in a covariate matched sample.

**Conclusion:**

Results show variability in normative cognitive aging patterns between research study participants in United States and Korean imaging studies. *APOE ε4* was more influential in rates of decline among Korean participants. These findings underscore the importance of replicating findings, diversifying study populations, and of considering ethnic and racialized backgrounds when examining genetic risk and cognitive aging, as different populations may show distinct patterns of decline.